# Higher Blood Cadmium Concentration Is Associated With Increased Likelihood of Abdominal Aortic Calcification

**DOI:** 10.3389/fcvm.2022.870169

**Published:** 2022-04-26

**Authors:** Zheng Qin, Qiang Liu, Pengcheng Jiao, Jiwen Geng, Ruoxi Liao, Baihai Su

**Affiliations:** ^1^Department of Nephrology, National Clinical Research Center for Geriatrics, West China Hospital, Sichuan University, Chengdu, China; ^2^Med+ Biomaterial Institute of West China Hospital, West China School of Medicine of Sichuan University, Chengdu, China; ^3^Med-X Center for Materials, Sichuan University, Chengdu, China; ^4^Chengdu First People's Hospital, Chengdu, China; ^5^West China School of Medicine, West China Hospital of Sichuan University, Chengdu, China

**Keywords:** cadmium, abdominal aortic calcification, vascular calcification, NHANES, cross-sectional study

## Abstract

**Aims:**

This study aimed to evaluate the association between blood cadmium concentration (BCC) and abdominal aortic calcification (AAC) in adults aged ≥40 years in the United States.

**Methods:**

Data were obtained from the 2013–2014 National Health and Nutrition Examination Survey (NHANES). Participants without data about BCC and AAC scores were excluded. BCC was directly measured using inductively coupled plasma mass spectrometry (ICP–MS). AAC scores were quantified by the Kauppila scoring system, and severe AAC was defined as an AAC score >6. Weighted multivariable regression analysis and subgroup analysis were conducted to explore the independent relationship between cadmium exposure with AAC scores and severe AAC.

**Results:**

A total of 1,530 participants were included with an average BCC of 0.47 ± 0.02 μg/L and AAC score of 1.40 ± 0.10 [mean ± standard error (SE)]. The prevalence of severe AAC was 7.96% in the whole subjects and increased with the higher BCC tertiles (Tertile 1: 4.74%, Tertile 2: 9.83%, and Tertile 3: 10.17%; *p* = 0.0395). We observed a significant positive association between BCC and the AAC score (β = 0.16, 95% *CI*: 0.01~0.30) and an increased risk of severe AAC [odds ratio (OR) = 1.45; 95% *CI*: 1.03~2.04]. Subgroup analysis and interaction tests revealed that there was no dependence for the association between BCC and AAC.

**Conclusion:**

Blood cadmium concentration was associated with a higher AAC score and an increased likelihood of severe AAC in adults in the United States. Cadmium exposure is a risk factor for AAC, and attention should be given to the management of blood cadmium.

## Introduction

Vascular calcification (VC) is the pathological deposition of calcium and phosphate minerals in the arterial walls of the cardiovascular system (1). Additionally, it is associated with abnormal intercellular signaling and failed anti-calcification mechanisms (2–4). Several studies have shown a significant more than 3-fold increase in the risk of cardiovascular events for patients with VC (5). However, there is currently no effective treatment for VC. Sodium thiosulfate alleviated the calcification progress in the iliac arteries and heart valves but failed to alleviate the abdominal aortic calcification (AAC) in patients with end-stage renal disease in a small double-blind, randomized, placebo-controlled study (6). Large-scale randomized controlled trials are still needed to confirm the potential of sodium thiosulfate to alleviate calcification (7). Thus, it is of great significance to explore the risk factors and prevent VC (8–10).

The abdominal aorta is a common site of VC, especially in patients with chronic kidney disease (CKD) (11). AAC has been widely recognized as an independent predictor of morbidity and mortality from cardiovascular events and as a reliable marker of both subclinical atherosclerotic disease and arteriosclerosis (12). To evaluate the degree of the calcified abdominal aorta, Kauppila et al. described a quantitative method for grading AAC (Kauppila AAC score) using lateral radiographs of the lumbar region (13). AAC score ranges from 0 to 24 and a higher AAC score corresponds to a more severe calcification condition. Due to its simplicity and high reproducibility, the Kauppila AAC score has been used widely in previous studies (14, 15).

Cadmium exposure is a serious global environmental problem that could lead to heavy health and socioeconomic burden (16). Cadmium can be commonly found in household garbage, industrial emissions of cadmium-containing substances, and even in the soil and water (17). The main causes of cadmium exposure include eating contaminated food, smoking, and working in cadmium-contaminated areas (18, 19). Previous studies have suggested that long-term cadmium exposure may increase the risk of developing coronary heart disease, hypertension, stroke, and heart failure (20, 21). A geometric mean cadmium level of 0.94 μg/g among 3,348 American Indian adults demonstrated a hazard ratio (HR) of 1.43 (95% *CI*: 1.21~1.70) for cardiovascular mortality and 1.34 (95% *CI*: 1.10~1.63) for coronary heart disease mortality (compared with the 80th to the 20th percentile of urine cadmium concentrations) in a prospective cohort study (22). Fagerberg et al. found that cadmium levels could be an independent risk factor for atherosclerotic plaques (23). Eum et al. reported that the blood cadmium concentration (BCC) was significantly higher among hypertensive patients than those without hypertension (1.77 vs. 1.64 mg/dl) and suggested that cadmium exposure might increase the blood pressure in the general Korean population (24). However, the exact range of blood cadmium that is associated with cardiovascular disease has not reached a consensus in previous studies.

The relationship between cadmium exposure and AAC has not been reported before. Thus, using the National Health and Nutrition Examination Survey (NHANES) cohort 2013–2014, we evaluated the association between BCC and AAC. We hypothesized that cadmium exposure was associated with an increased likelihood of AAC.

## Materials and Methods

### Study Population

Cross-sectional data were obtained from NHANES, a national cross-sectional study designed to assess the health and nutrition status of the U.S. general population administered by the National Center for Health Statistics (NCHS) through interviews and examinations. Due to the stratified multistage probability sampling method applied in the NHANES study design, the included samples showed relatively high representativeness. All NHANES data used in our analysis are publicly available at https://www.cdc.gov/nchs/nhanes/.

**Figure 1:**
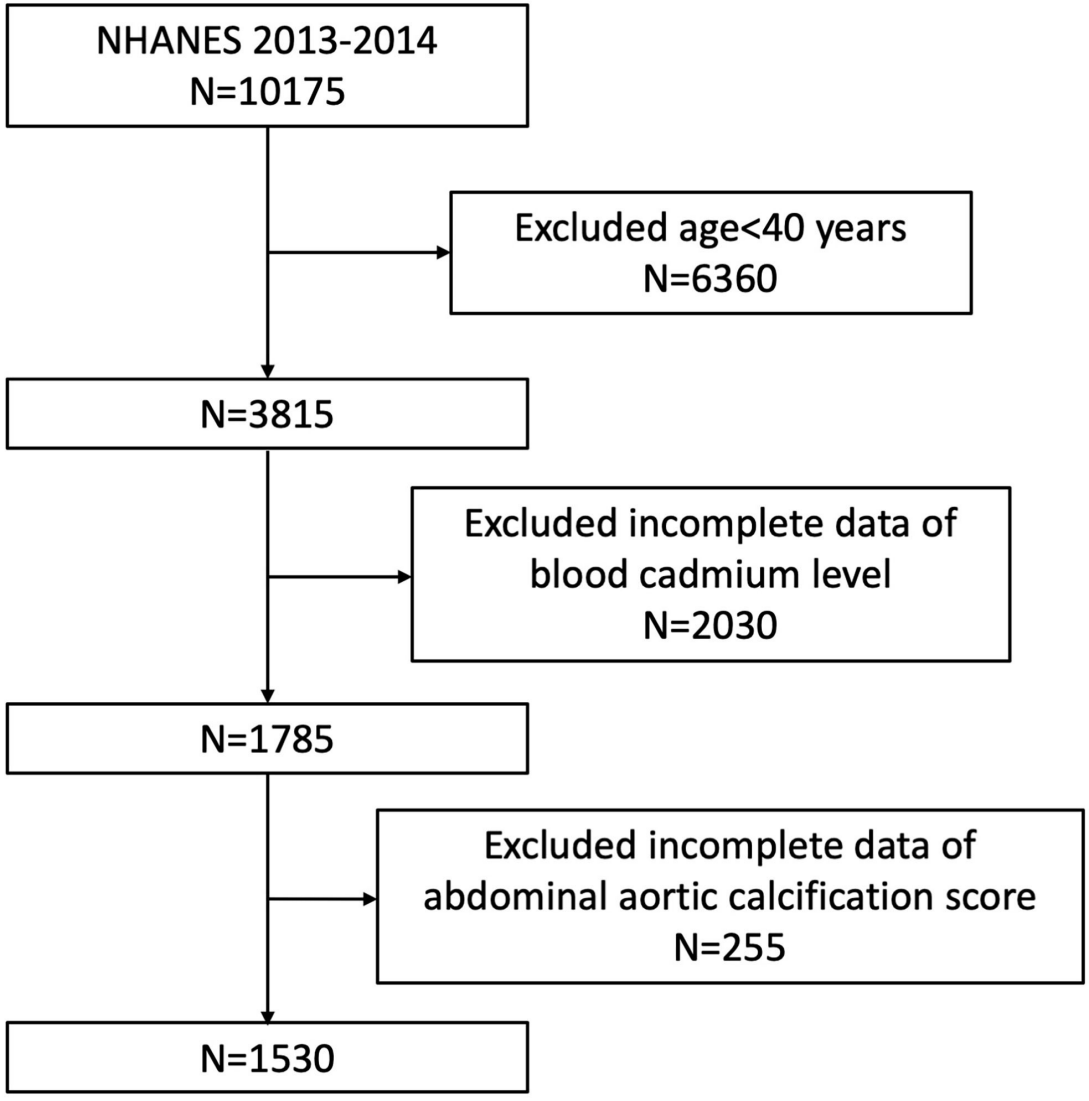
Flowchart of the sample selection from the 2013–2014 National Health and Nutrition Examination Survey (NHANES).

To explore the possible association between BCC and AAC, our analysis was based on the 2013–2014 NHANES, since only this cycle includes data on BCC and AAC scores. In addition, we excluded participants younger than 40 years because the AAC scores were obtained by dual-energy X-ray absorptiometry (DXA) scans while they did not undergo DXA in 2013–2014 according to the NHANES. The analytic cohort was restricted to subjects aged ≥ 40 years with complete blood cadmium and AAC scores data. A total of 10,175 samples were enrolled at first. Then, 1,530 participants were selected as the final analysis sample after excluding people under 40 years old (*n* = 6,360), incomplete BCC data (*n* = 2030), or AAC score (*n* = 255) (Figure 1).

The NCHS Ethics Review Board granted human subject approval for the conduction and study protocol of NHANES. Written informed consent was obtained from all participants.

### Exposure and Outcome Definitions

The blood cadmium level was designed as an exposure variable. Whole blood samples were collected by phlebotomists and the cadmium concentration in whole blood was measured directly using inductively coupled plasma mass spectrometry (ICP–MS, ELAN®DRC II, PerkinElimer, Norwalk) after a simple dilution sample preparation step as previously described (25). The detailed laboratory steps and methods of blood sample collection and processing are publicly available at https://wwwn.cdc.gov/nchs/data/nhanes/2013-2014/labmethods/PbCd_H_MET.pdf. BCC was calculated in a single measurement. The limit of detection (LOD) for cadmium was 0.10–0.20 μg/L. Results less than the detection range were assigned a value of LOD divided by the square root of two in the NHANES study design. It was noted that BCC was log 2-transformed when conducting regression analysis because they were right-skewed distributed. There was no missing value for blood cadmium in our analysis.

The AAC score and severe AAC were designed as outcome variables. The AAC score was quantified by evaluating lateral lumbar spine images obtained from DXA (Densitometer Discovery A, Hologic, Marlborough, MA, USA) performed by trained NHANES affiliated staff from a single measurement in a mobile examination center (MEC). The total AAC score for each participant was quantified strictly according to the Kauppila scoring method with a range from 0 to 24 by professional technologists, which has been widely reported and used in assessing the severity of calcified vessels before (13). A higher AAC score corresponded to a much more serious calcification condition of the abdominal aorta. According to previous studies, severe AAC was designed as another outcome variable as well; it was defined as a total AAC score >6, which represented significant aortic calcification lesions (26–28).

### Covariates

Based on a review of the literature, potential covariates that might confound the association between blood cadmium and AAC were summarized in our multivariable-adjusted models (29–31). Covariates in our study included gender (male/female), age (years), race (Mexican American/other Hispanic/non-Hispanic White/non-Hispanic Black/other races), education level (less than high school/high school or general educational development/above high school), smoker (yes/no), body mass index (BMI), systolic blood pressure (SBP), diastolic blood pressure (DBP), serum creatinine, estimated glomerular filtration rate (eGFR), alanine aminotransferase (ALT), aspartate aminotransferase (AST), hemoglobin A1c, serum uric acid, serum calcium, serum phosphorus, serum vitamin B12, total cholesterol, total 25-hydroxyvitamin D, hypertension, and diabetes status. Diabetes was defined as taking hypoglycemic medications or having a diagnosis of diabetes, a hemoglobin A1c level ≥ 6.5%, a fasting plasma glucose ≥126 mg/dl, or a 2-h plasma glucose ≥ 200 mg/dl (32). Hypertension was defined as taking antihypertensive medications, having a diagnosis of hypertension, or having three consecutive systolic blood pressure readings ≥140 mmHg or diastolic blood pressure ≥ 90 mmHg (33). Data about gender, race, age, and serum creatinine were used to calculate the estimated glomerular filtration rate (eGFR) according to the CKD Epidemiology Collaboration (CKD-EPI) creatinine equation (34). BMI was categorized as <25, 25–29.9, and ≥ 30 kg/m^2^, which corresponded to normal weight, overweight, and obese populations for all participants. All detailed measurement processes of the study variables are publicly available at www.cdc.gov/nchs/nhanes/.

### Statistical Analysis

Statistical analysis was conducted according to CDC guidelines, and appropriate NHANES sampling weights were applied and accounted for the complex multistage cluster survey design in the analysis.

Continuous variables were presented as the mean with standard error (SE), and categorical variables were presented as percentages. Either a weighted Student's *t*-test (for continuous variables) or a weighted chi-square test (for categorical variables) was used to evaluate the differences in groups divided by BCC (tertiles). BCC was right-skewed distributed and log 2-transformed when conducting regression analysis. A Pearson correlation analysis was performed to test the relationship between covariates and BCC. Multivariate logistic regression models were employed to explore the independent relationship between BCC and ACC (including AAC score and severe AAC) in three different models. In Model 1, no covariates were adjusted. Model 2 was adjusted for gender, age, and race. Model 3 was adjusted for gender, age, race, education level, BMI, SBP, DBP, serum creatinine, eGFR, ALT, AST, serum cotinine, hemoglobin A1c, serum uric acid, serum calcium, serum phosphorus, serum vitamin B12, total cholesterol, total 25-hydroxyvitamin D, folic acid intake, hypertension, and diabetes status. In addition, BCC was further converted from a continuous variable to a categorical variable (tertiles) for sensitivity analysis. Subgroup analysis stratified by gender, age, BMI (normal weight, overweight, and obese), hypertension, and diabetes was also performed using stratified multivariate regression analysis. In addition, an interaction term was added to test the heterogeneity of associations between the subgroups. The value of *p* < 0.05 was considered statistically significant. All analyses were performed using Empower software (www.empowerstats.com; X&Y solutions, Inc., Boston MA) and R version 4.1.2 (http://www.R-project.org, The R Foundation).

## Results

### Baseline Characteristics of the Enrolled Participants

The weighted baseline characteristics of the included individuals are shown in Table 1. Our analysis enrolled a total of 1,530 participants with a mean age of 57.29 ± 0.37 years old, of whom 48.61% were men and 51.39% were women. The mean BCC was 0.47 ± 0.02 μg/L overall, and the ranges of BCC tertiles 1–3 were 0.07–0.24, 0.25–0.50, and 0.52–7.32 μg/L, respectively. The overall mean AAC score was 1.40 ± 0.10, and participants in higher BCC tertiles tended to show higher AAC scores (Tertile 1: 0.98 ± 0.13, Tertile 2: 1.64 ± 0.20, and Tertile 3: 1.67 ± 0.26; *p* = 0.0398). The prevalence of severe AAC score was found in 7.96% of the whole subjects and increased with the higher BCC tertiles as well (Tertile 1: 4.74%, Tertile 2: 9.83%, and Tertile 3: 10.17%; *p* = 0.0395). Among different BCC tertiles, we found significant differences in age, gender, race, educational level, smoking, hypertension, BMI, DBP, hypertension status, serum calcium, serum phosphorus, and total cholesterol (all *p* < 0.05). There was no significant difference in diabetes, SBP, serum creatinine, eGFR, ALT, AST, hemoglobin A1c, serum uric acid, serum vitamin B12, or total 25-hydroxyvitamin D (all *p* > 0.05). The results for the association of covariates with BCC are shown in Supplementary Table 1. Gender, race, smoking status, diabetes, serum phosphorus, and total cholesterol were positively correlated with BCC. Educational level, hypertension, serum uric acid, and total 25-hydroxyvitamin D showed negative correlations with BCC. These covariables were all summarized in our further multivariable-adjusted regression models.

**Table 1:** Weighted baseline characteristics of participants according to blood cadmium levels.

	**Overall**	**Tertile 1** **(0.07–0.24)**	**Tertile 2** **(0.25–0.50)**	**Tertile 3** **(0.51–7.23)**	***P*-value**
	***n* = 1,530**	***n* = 496**	***n* = 522**	***n* = 512**	
Age (year)	57.29 ± 0.37	54.91 ± 0.55	59.71 ± 0.88	57.63 ± 0.59	0.0003
**Gender, % (SE)**					
Male	48.61 (1.01)	63.18 (2.54)	41.59 (2.33)	36.80 (3.37)	<0.0001
Female	51.39 (1.01)	36.82 (2.54)	58.41 (2.33)	63.20 (3.37)	
**Race, % (SE)**					
Mexican American	7.22 (1.70)	8.53 (2.04)	6.96 (1.51)	5.68 (2.02)	<0.0001
Other Hispanic	4.52 (0.94)	4.87 (0.98)	4.66 (1.20)	3.87 (1.30)	
Non-Hispanic White	71.02 (3.34)	75.11 (3.23)	72.65 (3.46)	63.24 (5.24)	
Non-Hispanic Black	10.23 (1.49)	8.45 (1.54)	8.74 (1.31)	14.57 (2.80)	
Other Races	7.01 (0.91)	3.04 (0.42)	6.98 (1.05)	12.64 (2.36)	
**Education level, % (SE)**					<0.0001
Less than high school	14.87 (1.82)	11.51 (2.19)	12.35 (1.72)	22.74 (3.43)	
High school or GED	21.91 (1.53)	24.62 (2.80)	15.39 (1.33)	26.17 (2.93)	
Above high school	63.22 (2.48)	63.86 (3.65)	72.26 (2.00)	51.09 (3.29)	
Smoker, % (SE)	45.92 (2.41)	27.98 (3.81)	40.95 (2.21)	77.32 (3.50)	<0.0001
Diabetes, % (SE)	12.59 (1.00)	14.23 (2.19)	12.85 (1.53)	9.97 (1.21)	0.1383
Hypertension, % (SE)	43.57 (1.53)	39.60 (3.19)	44.09 (2.07)	48.52 (2.72)	0.0221
BMI (kg/m2)	28.53 ± 0.16	29.35 ± 0.31	28.47 ± 0.18	27.45 ± 0.26	0.0009
SBP (mmHg)	125.11 ± 0.76	124.70 ± 1.05	125.17 ± 1.10	125.62 ± 1.40	0.6229
DBP (mmHg)	70.79 ± 0.49	72.66 ± 0.57	69.70 ± 0.72	69.48 ± 0.94	0.0111
Serum creatinine (mg/dL)	0.93 ± 0.02	0.94 ± 0.02	0.92 ± 0.02	0.94 ± 0.03	0.9549
eGFR (ml/min/1.73m2)	84.55 ± 0.72	86.43 ± 1.07	82.73 ± 1.26	84.12 ± 1.62	0.1935
ALT (IU/L)	24.92 ± 0.66	26.28 ± 0.68	23.81 ± 0.96	24.34 ± 1.38	0.1423
AST (IU/L)	25.36 ± 0.46	25.16 ± 0.37	25.39 ± 0.86	25.61 ± 1.00	0.6919
Hemoglobin A1c (%)	5.74 ± 0.03	5.75 ± 0.06	5.75 ± 0.04	5.72 ± 0.06	0.8002
Serum uric acid (μmol/L)	319.25 ± 2.40	324.26 ± 3.80	320.19 ± 4.61	310.83 ± 6.28	0.0866
Serum calcium (mmol/L)	2.37 ± 0.00	2.36 ± 0.00	2.36 ± 0.01	2.38 ± 0.01	0.0417
Serum phosphorus (mmol/L)	1.23 ± 0.01	1.22 ± 0.01	1.22 ± 0.01	1.26 ± 0.01	0.0354
Serum vitamin B12 (pmol/L)	476.07 ± 8.41	479.99 ± 17.83	483.95 ± 14.48	460.46 ± 22.58	0.5663
Total cholesterol (mmol/L)	5.10 ± 0.02	5.00 ± 0.06	5.13 ± 0.08	5.20 ± 0.04	0.0173
Total 25-hydroxyvitamin D (nmol/L)	75.88 ±1.43	75.34 ± 1.93	79.50 ± 1.36	72.13 ± 2.39	0.3281
Blood cadmium level (μg/L)	0.47 ± 0.02	0.16 ± 0.00	0.35 ± 0.00	1.06 ± 0.06	<0.0001
AAC score	1.40 ± 0.10	0.98 ± 0.13	1.64 ± 0.20	1.67 ± 0.26	0.0398
Severe AAC, % (SE)	7.96 (0.86)	4.74 (1.28)	9.83 (1.75)	10.17 (1.96)	0.0395

*Values are presented as the mean ± standard error (SE) unless stated otherwise*.

*GED, general educational development; BMI, body mass index; SBP, systolic blood pressure; DBP, diastolic blood pressure; eGFR, estimated glomerular filtration rate; ALT, alanine aminotransferase; AST, aspartate aminotransferase; AAC, abdominal aortic calcification*.

### Higher BCC Is Associated With a Higher AAC Score

We observed a significant positive association between BCC and AAC score (Model 1: β = 0.25, 95% *CI*: 0.12~0.39; Model 2: β = 0.19, 95% *CI*: 0.07~0.32; and Model 3: β = 0.16, 95% *CI*: 0.01~0.30). In the fully adjusted model (Model 3), a 1-unit increase in log 2-transformed blood cadmium levels was associated with a 0.16-unit higher AAC score.

To further assess the association between BCC and AAC scores, we converted BCC from continuous variables to categorical variables (tertiles). In the fully adjusted model (Model 3), the β in the highest tertile (Tertile 3) when compared with the lowest tertile (Tertile 1) was 0.30 (95% *CI*: −0.11~0.71). The average AAC score of Tertile 2 was 0.16 per unit higher than that of Tertile 1, though this difference was not statistically significant (95% *CI*: −0.21~0.53) (Table 2).

**Table 2:** Multivariate logistic regression models of AAC score with blood cadmium levels.

**Blood cadmium level**	**β^a^ **(95% CI**^b^)**		
	**Model 1^**c**^**	**Model 2^**d**^**	**Model 3^**e**^**
Continuous	0.25 (0.12, 0.39)	0.19 (0.07, 0.32)	0.16 (0.01, 0.30)
**Categories**			
Tertile 1	Reference	Reference	Reference
Tertile 2	0.66 (0.29, 1.03)	0.17 (−0.18, 0.52)	0.16 (−0.21, 0.53)
Tertile 3	0.69 (0.30, 1.08)	0.46 (0.08, 0.83)	0.30 (−0.11, 0.71)

*In sensitivity analysis, blood cadmium level was converted from a continuous variable to a categorical variable (tertiles)*.

*^a^Effect size;*

*^b^95% CI: 95% confidence interval;*

*^c^Model 1: no covariates were adjusted;*

*^d^Model 2: adjusted for gender, age, and race;*

*^e^Model 3: adjusted for gender, age, race, education level, BMI, SBP, DBP, serum creatinine, eGFR, ALT, AST, hemoglobin A1c, serum uric acid, serum calcium, serum phosphorus, serum vitamin B12, total cholesterol, total 25-hydroxyvitamin D, smoking, hypertension, and diabetes status*.

**Figure 2:**
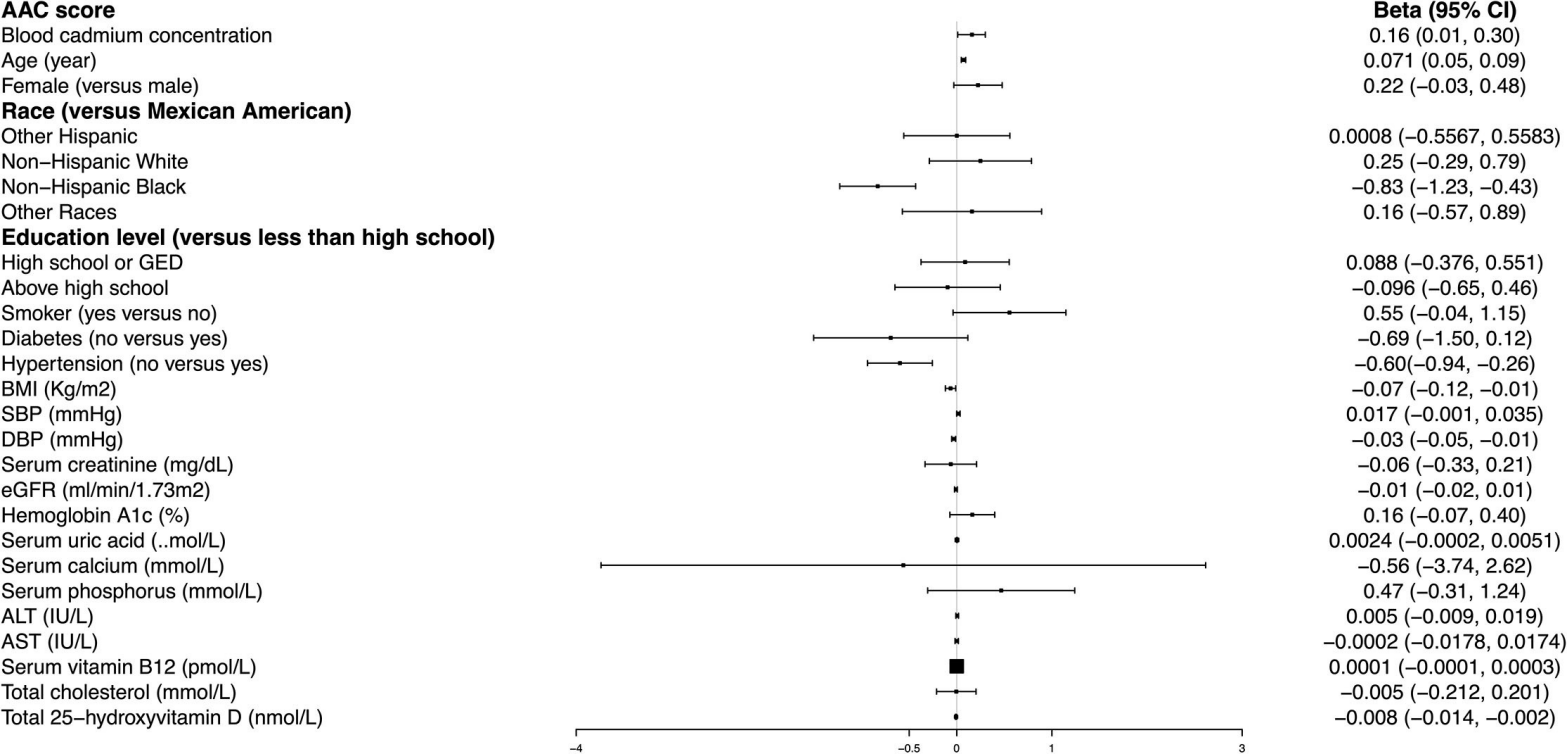
Multivariate logistic regression models of abdominal aortic calcification (AAC) score.

Age, hypertension, and BMI remained significantly associated with the AAC score in the fully adjusted model. Compared with those participants with hypertension, non-hypertension subjects showed 0.60 lower AAC scores (95% *CI*: −0.94– −0.26). Each increased unit in age and BMI was associated with 0.071 higher and 0.07 lower AAC scores, respectively (Figure 2).

### Higher BCC Is Associated With an Increased Risk of Severe AAC

A significant positive association was found between BCC and the occurrence of severe AAC. In Model 1, the unadjusted odds ratio (*OR*) was 1.36 (95% *CI*: 1.07~1.73). In Model 2 adjusted for age, gender, and race, the adjusted *OR* was 1.59 (95% *CI*: 1.09~2.31). In Model 3 adjusted for all potential covariables, the fully-adjusted *OR* was 1.45 (95% *CI*: 1.03~2.04), indicating that each unit of increased log 2-transformed BCC was associated with a 45% increased risk of severe AAC. In the sensitivity analysis that treated BCC in tertiles, the fully-adjusted *OR* (reference to Tertile 1) was 1.89 (95% *CI*: 0.74, 4.80) for Tertile 2 and 1.84 (95% *CI*: 0.58~5.85) for Tertile 3. Our results suggested that higher BCC was associated with an increased risk of severe AAC (Table 3).

**Table 3:** Multivariate logistic regression models of the AAC group with blood cadmium levels (severe AAC vs. no AAC).

**Blood cadmium level**	**OR^a^ **(95% CI**^b^)**		
	**Model 1^**c**^**	**Model 2^**d**^**	**Model 3^**e**^**
**Severe AAC** ^**f**^			
Continuous	1.36 (1.07, 1.73)	1.59 (1.09, 2.31)	1.45 (1.03, 2.04)
**Categories**			
Tertile 1	Reference	Reference	Reference
Tertile 2	2.65 (1.31, 5.37)	1.86 (0.86, 4.03)	1.89 (0.74, 4.80)
Tertile 3	2.70 (1.12, 6.49)	2.56 (0.90, 7.29)	1.84 (0.58, 5.85)

*In sensitivity analysis, blood cadmium level was converted from a continuous variable to a categorical variable (tertiles)*.

*^a^OR: odds ratio*.

*^b^95% CI: 95% confidence interval*.

*^c^Model 1: no covariates were adjusted*.

*^d^Model 2: adjusted for gender, age, and race*.

*^e^Model 3: adjusted for gender, age, race, education level, BMI, SBP, DBP, serum creatinine, eGFR, ALT, AST, hemoglobin A1c, serum uric acid, serum calcium, serum phosphorus, serum vitamin B12, total cholesterol, total 25-hydroxyvitamin D, smoke, hypertension, and diabetes status*.

*^f^AAC: abdominal aortic calcification*.

**Figure 3:**
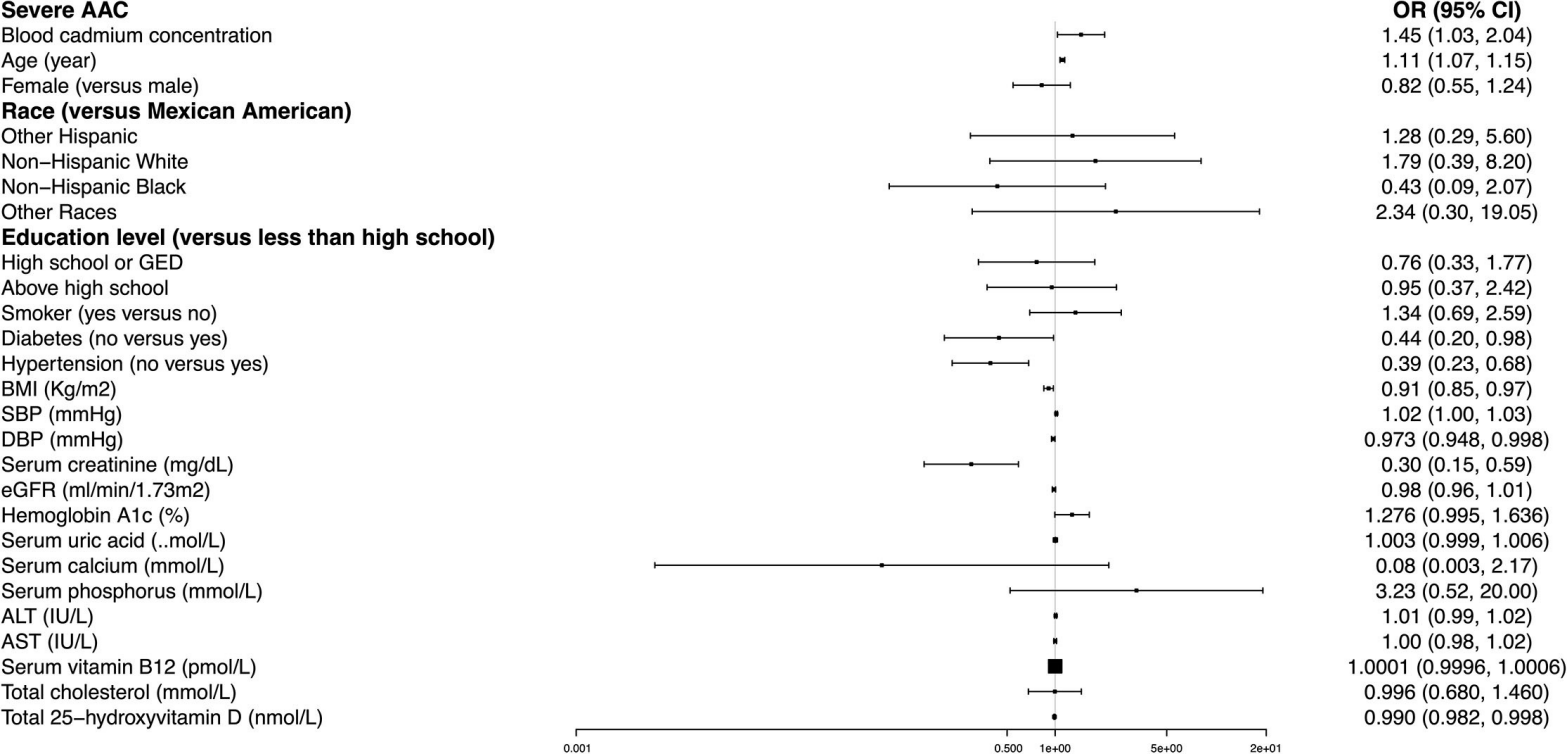
Multivariate logistic regression models of severe AAC.

In our fully adjusted model, age, diabetes, hypertension, BMI, SBP, DBP, serum creatinine, and total 25-hydroxyvitamin D remained significantly associated with the odds of severe AAC. Non-hypertensive and non-diabetic subjects had 61% (*OR* = 0.39, 95% *CI*: 0.23~0.68) and 56% (*OR* = 0.44, 95% *CI*: 0.20~0.98) lower likelihoods of having severe AAC than their counterparts. Each increased unit in age and SBP was associated with an 11 and 2% higher likelihood, and each higher unit in BMI, DBP, serum creatinine, and total 25-hydroxyvitamin D corresponded to a 9, 2.7, 70, and 1% lower risk of severe AAC, respectively (Figure 3).

### Subgroup Analysis

**Figure 4:**
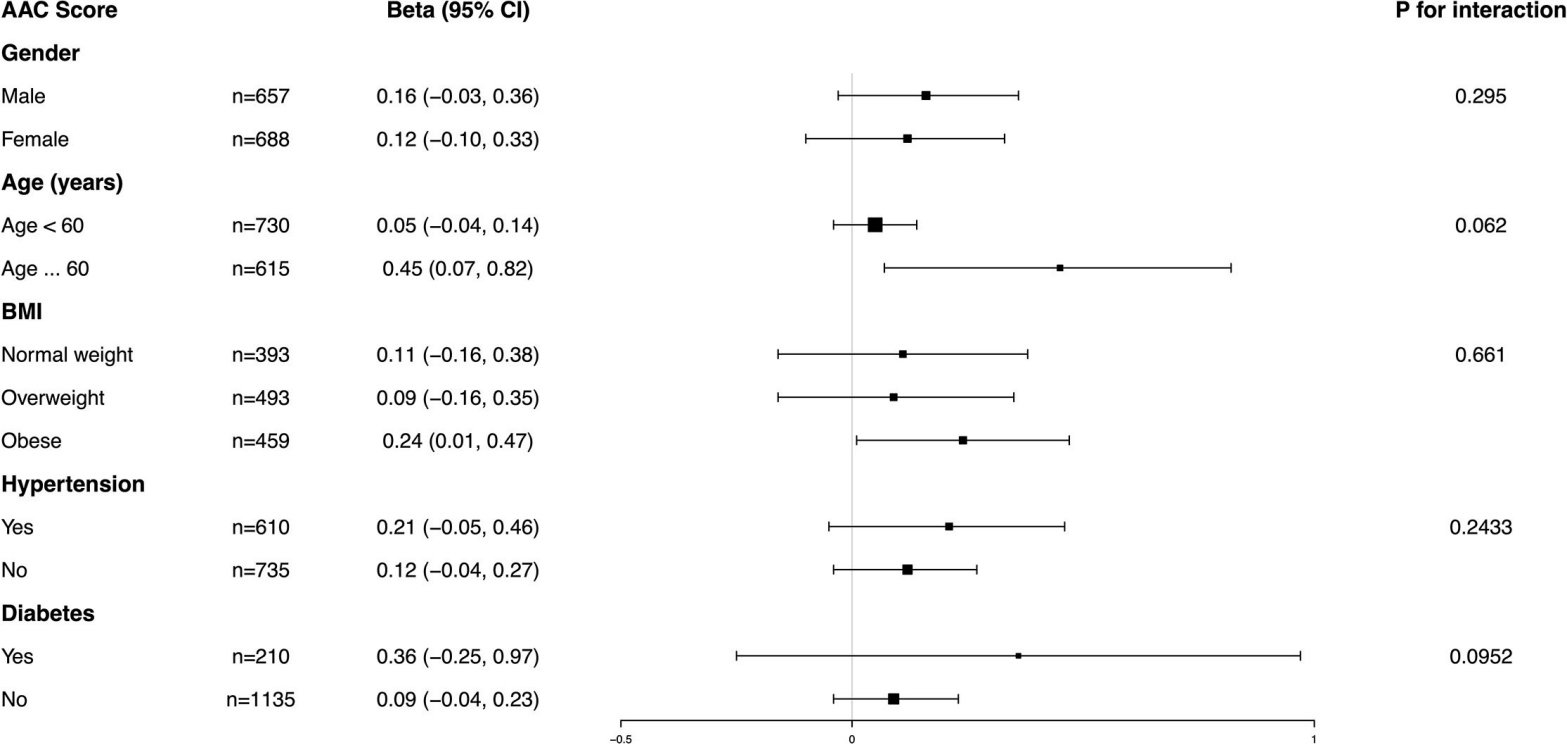
Subgroup analysis for the association between blood cadmium concentration (BCC) and AAC score.

**Figure 5:**
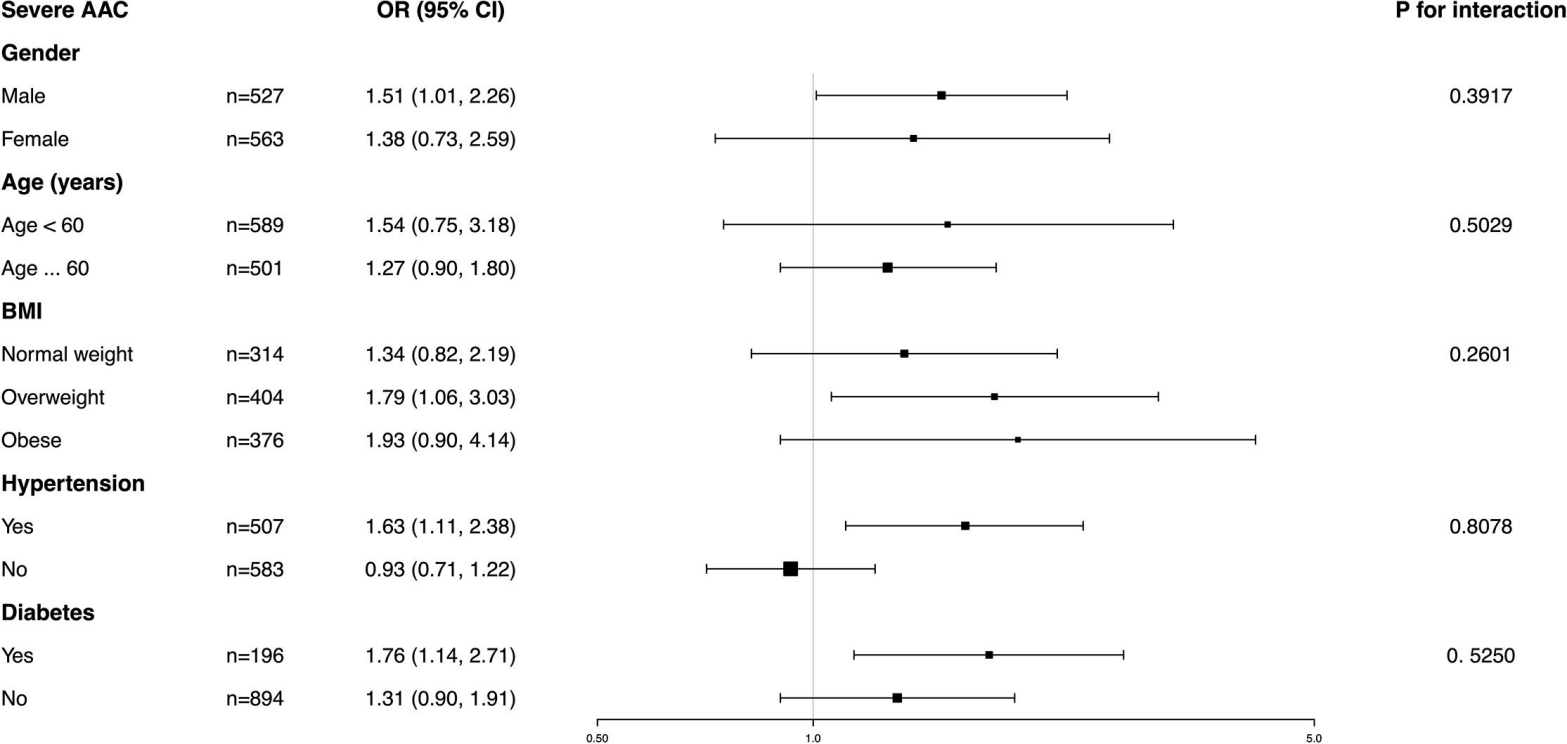
Subgroup analysis for the association between BCC and severe AAC.

Subgroup analysis was performed to further evaluate the robustness of the association between BCC and AAC. In addition, an interaction test of gender, age, BMI, hypertension, and diabetes was conducted. However, no correlation with the *p* for interaction meeting the statistical significance was detected, indicating that there was no dependence on gender, age, BMI, hypertension, or diabetes for this association (all *p* for interaction >0.05) (Figures 4, 5).

It was noted that BCC positively correlated with a higher AAC score in the obese population (β = 0.24, 95% *CI*: 0.01~0.47) and an increased risk of severe AAC in overweight individuals (*OR* = 1.93, 95% *CI*: 0.90~4.14), while in the normal weight population, this association was not statistically significant, indicating that this association could be more significant in overweight and obese individuals. However, a value of *p* for the interaction of 0.6610 for the AAC score and 0.2601 for severe AAC suggested that BMI showed no dependence on the association of BCC and AAC. Our results from the subgroup analysis suggest that this positive correlation is similar in differents population settings.

## Discussion

In this cross-sectional study of 1,530 participants, we observed that participants with higher BCC exhibited higher AAC scores and an increased risk of severe AAC. Subgroup analysis indicated that this association was similar in different populations with gender, age, BMI, hypertension, and diabetes status. Our findings suggest that cadmium exposure and blood cadmium burden should be considered for people with AAC in clinical settings.

To the best of our knowledge, this is the first study assessing the association between BCC and AAC. Several studies have demonstrated a positive association between BCC and cardiovascular diseases, such as dilated cardiomyopathy, coronary heart disease, stroke, and peripheral arterial disease (35–37). In a study exploring the relationship between cadmium exposure and coronary atherosclerosis, BCC was positively correlated with the severity of coronary atherosclerosis (38). Anurag et al. found that urinary cadmium concentration was associated with an ankle-brachial index >1.4 in at least one leg, which was thought to be associated with vascular incompressibility due to the calcification of the arterial walls (39). Eum et al. reported that participants with hypertension had higher BCC than those without hypertension (1.77 vs. 1.64 mg/dl) (24). However, the exact range of blood cadmium that is considered safe has not been reported to reach a consensus. Previous studies suggested that although only 1–5% of ingested and 25–50% of inhaled cadmium was absorbed, its biological half-life in the human body was exceptionally long (15–45 years) (40). In addition, low-level cadmium exposure has been recognized to be associated with a decline in renal function and an independent risk factor for kidney disease, thus, the safe range of BCC is still undetermined (41). Our results are consistent with previous studies, suggesting a positive correlation between BCC and AAC, indicating that blood cadmium burden has a significant negative impact on cardiovascular health.

Although cadmium exposure has been recognized as a potential risk factor for cardiovascular disease, the underlying mechanisms remain unclear. One possible mechanism is by altering lipid profiles. Clinical studies have shown that the increased BCC is associated with dyslipidemia, such as high total cholesterol (TC), triglycerides (TGs), low-density lipoprotein cholesterol (LDL-C), and low high-density lipoprotein cholesterol (HDL-C) (42). Animal experiments revealed that cadmium significantly increased TC, TG, LDL-C, and free fatty acid levels in rats while decreasing HDL-C concentration (43). Besides, the altered lipid profile induced by cadmium, including increased cholesterol, lipotoxicity, and lipogenesis, contributes to the progression of VC (44, 45). Moreover, cadmium exposure could stimulate lipid deposition in the carotid artery and aorta (46, 47). Other possible mechanisms by which cadmium-induced calcification could occur include inflammation and oxidative stress. Studies have shown that cadmium could lead to the upregulation of mediators and markers of inflammation, such as interleukin (IL)-6, IL-8, C-reactive protein (CRP), and tumor necrosis factor-α (TNF-α), which appear to have pro-inflammatory properties (35, 48, 49). In addition, cadmium exposure was associated with increased plasma soluble urokinase plasminogen activator, an emerging inflammatory biomarker (50). Cadmium can promote oxidation by inducing redox-active species, which may contribute to lipid peroxidation and oxidative DNA damage (35, 38). Previous studies have found a significant increase in proinflammatory cytokines, such as IL-2, IL-6, and TNF-α in the cadmium exposure group, as well as a 2-fold increase in oxidative LDL levels (51). The cadmium-induced oxidative stress may be involved in the decreased activity of antioxidant glutathione and enzymes, such as paraoxonase 1 (PON1), which is thought to have anti-inflammatory and antioxidant effects (51, 52). In addition, oxidative stress induced by cadmium can lead to apoptosis, which may be related to the activation of nuclear factor-kappa B (NF-κB), a redox-sensitive transcription factor that plays an important role in regulating apoptosis (53). These elevated inflammatory cytokines and oxidative stress could also activate the receptor activator of nuclear factor-κB ligand (RANKL)/osteoprotegerin (OPG) system and upregulate the expression of bone morphogenetic proteins (BMPs), thus contributing to the osteoblast differentiation of vascular smooth muscle cells, which may be an intermediary mechanism associated with cadmium exposure as well (54, 55).

Our subgroup analysis found that the association between BCC and VC was more significant in the overweight and obese populations. Similar to our results, a study by Choi found an association between blood cadmium and osteoporosis in obese men, but not in non-obese men (56). Emily et al. found that cadmium exposure was associated with liver injury and systemic inflammation in the obesity subgroup, while there was no statistical significance in the non-obese subgroup (57). In a study by Alexandre, higher urinary cadmium levels were associated with significantly elevated blood pressure among the obese group, while no significant association was found in either the normal-weight or overweight group (58). Contrary to our results, Wang et al. found that the association between urinary cadmium and hypertension was significant in normal-weight participants but not in those who were overweight or obese (59). Although the exact mechanism was not fully elucidated, a possible explanation may be the poorer lipid profiles and the increased oxidative stress caused by obesity and cadmium exposure. Longitudinal studies have confirmed a correlation between obesity and increased oxidative stress (60). The VC process may be accelerated additively and synergistically by the simultaneous cadmium exposure and obesity-induced worse lipid profile and oxidative stress.

Our research has several strengths. First, our study was based on data from NHANES, a nationwide, population-based sample obtained using a standard protocol. In addition, we adjusted for confounding covariates, and the selection of covariates was mainly based on previous studies that assessed the relationship of AAC with other exposure variables to ensure that our results were reliable. However, limitations cannot be ignored. Due to the cross-sectional study design, we could not obtain a causal relationship between BCC and AAC. Based on our results, we suggest that higher BCC may increase the risk of AAC, but we cannot further reveal the exact BCC range that was associated with AAC. Although we adjusted for some potential covariates, we could not completely rule out the influence of other possible confounding factors, such as diuretics, hormones, and drug use, which may influence calcification. In addition, participants in our analysis were enrolled from a single country, and their ethnicity may not be applicable to many countries around the world. Exposure to cadmium may also vary within and between countries. Our study cannot reflect a worldwide or multi-ethnic cohort, thus limiting the ability to generalize our findings to a general population or other ethnic groups.

## Conclusion

Our study demonstrated that increased blood cadmium levels were associated with an increased AAC score and a risk of severe AAC in adults aged ≥ 40 years in the United States, indicating that cadmium exposure has a negative effect on cardiovascular health. However, further studies are still needed to validate our findings.

## Data Availability Statement

Publicly available datasets were analyzed in this study. This data can be found at: https://www.cdc.gov/nchs/nhanes/.

## Ethics Statement

The studies involving human participants were reviewed and approved by the NCHS Ethic Review Board. The patients/participants provided their written informed consent to participate in this study.

## Author Contributions

ZQ: data analysis, formal analysis, methodology, and writing—original draft. QL: software and writing—original draft. PJ: writing—original draft. JG: data analysis. RL: software and funding acquisition. BS: conceptualization, funding acquisition, and writing—review and editing. All the authors approved the final version.

## Funding

This work was supported by the National Natural Science Foundation of China (Grant No. 82000702), the Sichuan Science and Technology Program (Grant No. 2022YFS0147), the Science and Technology Achievement Transformation Fund of West China Hospital of Sichuan University (Grant No. CGZH19006), the Med-X Innovation Programme of Med-X Center for Materials of Sichuan University (Grant No. MCM202101), the 1.3.5 project for disciplines of excellence from West China Hospital of Sichuan University (Grant No. ZYJC21010), and Med+ Biomaterial Institute of West China Hospital/West China School of Medicine of Sichuan University (Grant No. ZYME20001).

## Conflict of Interest

The authors declare that the research was conducted in the absence of any commercial or financial relationships that could be construed as a potential conflict of interest.

## Publisher's Note

All claims expressed in this article are solely those of the authors and do not necessarily represent those of their affiliated organizations, or those of the publisher, the editors and the reviewers. Any product that may be evaluated in this article, or claim that may be made by its manufacturer, is not guaranteed or endorsed by the publisher.
