# Supplementary material for: Higher Blood Cadmium Concentration Is Associated With Increased Likelihood of Abdominal Aortic Calcification
Source: Front Cardiovasc Med. 2022 Apr 26;9:870169. doi: 10.3389/fcvm.2022.870169 (PMC9086707; doi:10.3389/fcvm.2022.870169)
Supplement: Supplementary file 1 [file Table_1.DOCX]

Supplementary Material

**Supplementary Table 1. Association of covariates with blood cadmium concentration in Pearson correlation analysis.**

|  | r | P value |
| --- | --- | --- |
| Age (years) | -0.02 | 0.4630 |
| Gender | 0.12 | <0.0001 |
| Race | 0.17 | <0.0001 |
| Education level | -0.18 | <0.0001 |
| Smoker | 0.38 | <0.0001 |
| Diabetes | 0.06 | 0.0254 |
| Hypertension | -0.07 | 0.0042 |
| BMI (Kg/m2) | -0.12 | <0.0001 |
| SBP (mmHg) | 0.01 | 0.6395 |
| DBP (mmHg) | -0.05 | 0.0680 |
| Serum creatinine (mg/dL) | -0.02 | 0.4994 |
| eGFR (ml/min/1.73m2) | 0.02 | 0.4296 |
| ALT (IU/L) | -0.02 | 0.3572 |
| AST (IU/L) | 0.04 | 0.1123 |
| Hemoglobin A1c (%) | -0.02 | 0.5445 |
| Serum uric acid (μmol/L) | -0.08 | 0.0027 |
| Serum calcium (mmol/L) | 0.05 | 0.0594 |
| Serum phosphorus (mmol/L) | 0.05 | 0.0435 |
| Serum vitamin B12 (pmol/L) | -0.01 | 0.6200 |
| Total cholesterol (mmol/L) | 0.08 | 0.0011 |
| Total 25-hydroxyvitamin D (nmol/L) | -0.07 | 0.0048 |

Abbreviations: BMI, body mass index; SBP, systolic blood pressure; DBP, diastolic blood pressure; eGFR, estimated-glomerular filtration rate; ALT, alanine aminotransferase; AST, aspartate aminotransferase; AAC, abdominal aortic calcification.
